# Immune Ablation and Stem Cell Rescue in Two Pediatric Patients with Progressive Severe Chronic Graft-Versus-Host Disease

**DOI:** 10.3390/ijms232315403

**Published:** 2022-12-06

**Authors:** Jaspar Kloehn, Anne Kruchen, Kerstin Schütze, Katharina Wustrau, Johanna Schrum, Ingo Müller

**Affiliations:** Division of Pediatric Stem Cell Transplantation and Immunology, Clinic of Pediatric Hematology and Oncology, University Medical Center Hamburg-Eppendorf, Martinistr. 52, 20246 Hamburg, Germany

**Keywords:** hematopoietic stem cell transplantation, graft-versus-host-disease, immunoablation

## Abstract

Transplantation of allogeneic hematopoietic stem cells represents an established treatment for children with high-risk leukemia. However, steroid-refractory chronic graft-versus-host disease (SR-cGvHD) represents a severe life-threatening complication, for which there is no standard therapy. After failing several lines of immunosuppressive and biological treatment, we applied an immunoablative therapy with re-transplantation of purified CD34^+^ donor stem cells to reset the aberrant immune system. Two pediatric patients, who had been transplanted for high-risk acute lymphoblastic leukemia, underwent the procedure. Interestingly, enough stem cells could be mobilized, harvested, and purified to be used as grafts more than one year after allogeneic transplantation under intensive immunosuppressive therapy and ongoing SR-cGvHD. With a follow-up of 8 and 22 months, respectively, both patients are without immunosuppressive therapy and do not show signs of active disease. Regeneration of skin manifestations started promptly, other damaged organs did not progress and continue to show recovery from severe fibrotic transformation. Bone marrow function is robust and T cell receptor repertoires showed polyclonal immune reconstitution. In conclusion, stem cell harvest and re-transplantation of human CD34^+^-selected allogeneic stem cells is possible and represents a new therapeutic option in SR-cGvHD by resetting a profoundly disturbed immune network.

## 1. Introduction

Chronic graft-versus-host disease (cGvHD) remains one of the leading causes of non-relapse morbidity and mortality in patients after allogeneic hematopoietic stem cell transplantation (HSCT) [1,2]. In pediatric patients with acute lymphoblastic leukemia (ALL), the incidence of cGvHD ranges between 10% and 20% [3]. Major factors in cGvHD pathogenesis are inflammation due to tissue damage, dysregulated T and B cells and tissue repair with fibrosis. According to the modified severity criteria of the National Institutes of Health (NIH), there are mild, moderate and severe courses [4]. The long-term mortality of severe cGvHD is approximately 50% [5]. Besides high mortality, cGvHD is associated with serious limitations in activities of daily life, continued need for immunosuppressive therapy, and organ dysfunction. In combination with joint and fascia manifestation, sclerodermatous cGvHD is one of the most disabling forms. Especially, pulmonary and hepatic cGvHD result in poor prognosis [6]. As first-line therapy, steroids and calcineurin inhibitors are used. Due to the lack of standard therapy for steroid-refractory cGvHD (SR-cGvHD), there is a plethora of salvage therapies [7]. Nonetheless, the prognosis of steroid-refractory cGvHD remains poor and the excessive use of steroids often adds to morbidity, e.g., by avascular necrosis of the bone [8]. Several molecular targets have been identified and precision medicines have been developed, thereby improved outcomes significantly, e.g., by the introduction of ruxolitinib [9].

In some severely affected patients after a long course of the cGvHD, the immunological network shows so many signs of malfunction that it seems inconceivable that a single drug is able to restore the physiological immune balance. In this situation, we hypothesized that a “reset” of the immune system might be a promising approach to overcome the arrested state of a weak, erroneous, misled immune system. To this end, all lymphocytes along with the immunological memory need to be eradicated. The concept of immune ablation by very lymphotoxic chemotherapy augmented by serological T cell depletion with antithymocyte immunoglobulin (ATG) has been used in some refractory autoimmune diseases, such as systemic sclerosis, dermatomyositis, multiple sclerosis, and others [10,11,12]. Bone marrow function is restored by a rescue with cryopreserved autologous stem cells in these diseases. In the present situation of cGvHD, the patients had undergone allogeneic HSCT already, and it was unclear if like in mice, human hematopoietic stem cells would engraft a second time, in particular after prolonged immune dysfunction and extensive immunosuppressive treatment. The severity of the SR-cGvHD in two children with ALL following allogeneic HSCT drove us to administer immunoablative therapy (IAT) and rescue hematopoiesis with mobilized, purified, previously transplanted hematopoietic stem cells in order to support regeneration of a naïve and self-tolerant immune system.

## 2. Results

### 2.1. Case 1

**Figure 1:**
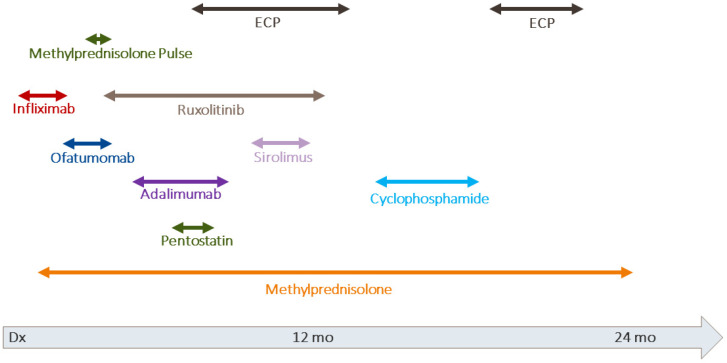
Treatment of cGvHD in case 1.

An 8 year old girl developed an early relapse of B precursor ALL. She underwent HSCT from a matched sibling donor. The conditioning regimen consisted of total body irradiation (TBI) and etoposide. GvHD prophylaxis cyclosporine A was used. Leukocyte engraftment was timely on day 12 and of complete donor origin. On day 25, she developed acute GvHD of the skin and received methylprednisolone. The patient showed an initial response, but required continued immune suppression. Thirteen months after HSCT, sclerodermatous cGvHD was diagnosed and histologically confirmed. In the further course, there were also other organ manifestations. According to the modified NIH criteria for organ scoring (with scores from 1 to 3) the maximum cGvHD activity was detected [5]. Skin, joints, and fascia were severely affected (score 3). The lungs showed moderate (score 2) and the eyes mild (score 1) symptoms. The overall grading resulted in severe cGvHD, which was steroid refractory. Despite the application of methylprednisolone pulses, infliximab, adalimumab, ofatumumab, ruxolitinib, pentostatin, sirolimus and extracorporeal photopheresis over a period of 2 years, the cGvHD progressed (Figure 1). Additional patient characteristics can be found can be found in Table S1. Facing the unfavorable prognosis of this severe treatment-refractory cGvHD, we resorted to an immune ablation with stem cell rescue as detailed below.

**Figure 2:**
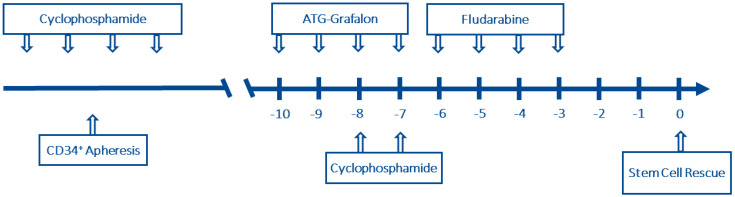
Immunoablative therapy (IAT) in both cases.

In preparation for stem cell apheresis, a dose of 2 g/m^2^ of cyclophosphamide was used for chemomobilization. Ten days after the administration of cyclophosphamide a dose of 15 µg/kg/d of granulocyte colony-stimulating factor (GCSF) was applied for 5 days. Afterwards, the patient underwent one stem cell apheresis with a yield of 2.05 × 10^6^/kg CD34^+^ cells after immunomagnetic purification using the CliniMacs^®^ System. These cells were cryopreserved. Due to subjective and minor clinical improvement, we administered three further pulses of cyclophosphamide at a dose of 1.5 g/m^2^ at intervals of three weeks. The immune ablation consisted of antithymocyte globulin Grafalon^®^ (4 × 15 mg/kg), cyclophosphamide (2 × 60 mg/kg) and fludarabine (4 × 40 mg/m^2^) as depicted in Figure 2. Weekly virus monitoring for HSV, CMV, EBV, and AdV in conjunction with acyclovir prophylaxis and antimycotic preemptive treatment with caspofungin was established. GCSF was administered from day 5 after stem cell rescue. After autologous stem cell rescue with the complete product, the patient showed stable engraftment on day 9 and except for reactivation of cytomegalovirus, no transplant-related complications were encountered. There was a complete response according to the modified NIH criteria for organ scoring except for lung scoring (score 2, which presented as terminal fibrotic lung injury without progression in the absence of immune suppression). Immunosuppressive treatment (cyclosporine A) was discontinued on day 203. There was an improvement in forced expiratory volume in the further course and the limited lung function status can be considered as residuum of cGvHD. Furthermore, osteonecrosis occurred as a side effect of long-term therapy with steroids prior to autologous stem cell transplantation. Nevertheless, the manifest improvement in our patient was consistent. The life-threatening SR cGvHD has been stopped (last follow-up day 698 after stem cell rescue).

### 2.2. Case 2

**Figure 3:**
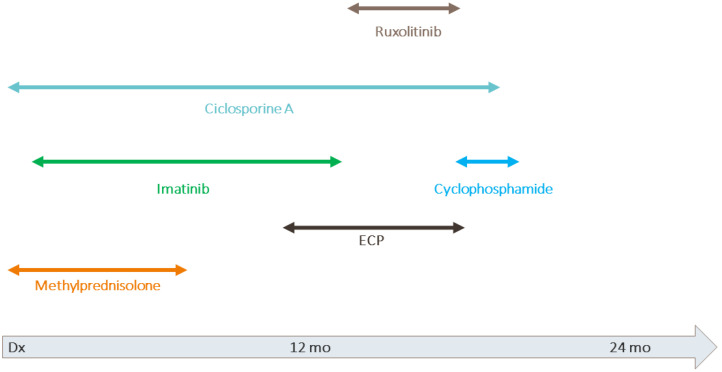
Treatment of cGvHD in case 2.

A 16-years-old boy was diagnosed with BCR/ABL positive common ALL and underwent HSCT from a matched unrelated donor. As a conditioning regimen, TBI, etoposide, and ATG was administered. Cyclosporine A and methotrexate were applied for GvHD prophylaxis. The patient engrafted on day 14 with complete donor chimerism. On day 16, acute skin GvHD occurred, which was successfully treated by the addition of methylprednisolone. While tapering immune suppression, six months after HSCT sclerodermatous cGvHD was diagnosed. More organs were affected in the further course. The overall grading based on the modified NIH criteria resulted in severe cGvHD activity (maximum scoring: skin: 3, joints and fascia: 3, lungs: 2, and eyes: 1). The patient did not respond to treatment with steroids. Subsequently, administration of methylprednisolone pulses, cyclosporine A, imatinib, ruxolitinib, and extracorporeal photopheresis remained ineffective and the cGvHD worsened (Figure 3). In analogy to case 1, we opted for immune ablation with stem cell rescue.

**Figure 4:**
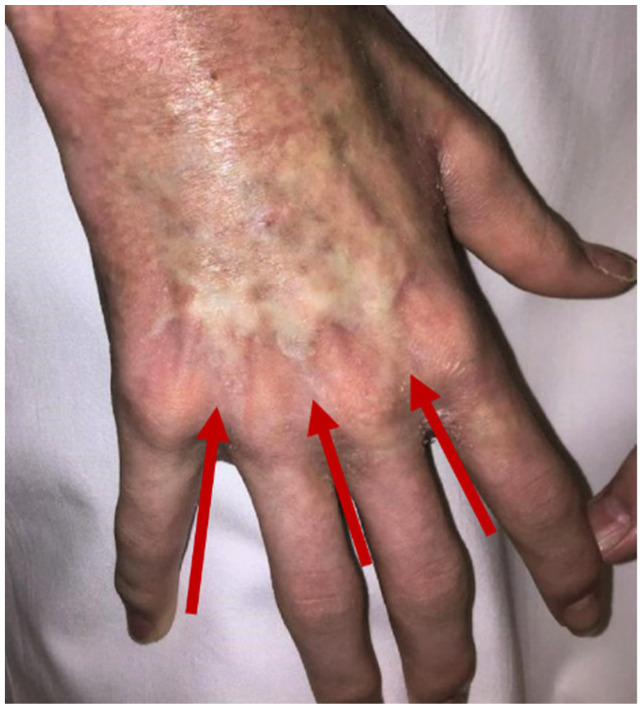
Regression of cGvHD in patient 2. Arrows indicate residual areas of fibrotic thickening of the skin, which had covered both hands and have resolved completely.

Chemomobilization was performed with 2 g/m^2^ of cyclophosphamide. Furthermore, GCSF with a dose of 15 µg/kg/d was used for 7 days. The stem cell apheresis was carried out on days 15 and 16 after the application of cyclophosphamide. Because of the clinical response, 3 further cyclophosphamide pulses were applied at intervals of 3 weeks (single dose of 1.5 g/m^2^). The weight-based dosing of immune ablation with antithymocyte globulin Grafalon^®^, cyclophosphamide, and fludarabine was identical to case 1. The transplanted CD34^+^ selected stem cells had a CD34^+^ cell content of 2.4 × 10^6^/kg while 2.7 × 10^3^/kg residual CD3^+^ T cells were detected. The patient tolerated our concept and engrafted on day 10 after stem cell rescue. As a transplantation-related complication, Epstein–Barr virus reactivation occurred. The strategy of immune ablation with stem cell rescue resulted in a partial response of cGvHD. There was a decrease in the skin as well as in joints and fascia score. The cGvHD activity of the eyes resolved while the lungs were still affected (NIH organ scoring: skin: 2, joints and fascia: 2, eyes: 0, and lungs: 2). The patient’s performance remained limited by the side effects of the previous therapy and by open wounds caused by regeneration of skin tissue. However, cGvHD progress has been stopped until today (last follow-up day 247 after stem cell rescue). Skin thickening retracted as shown in Figure 4. There was no need for significant immunosuppression. Only low dose methylprednisolone (0.1 mg/kg/d) was transiently applied.

### 2.3. Immune Reconstitution after Immunoablation

**Figure 5:**
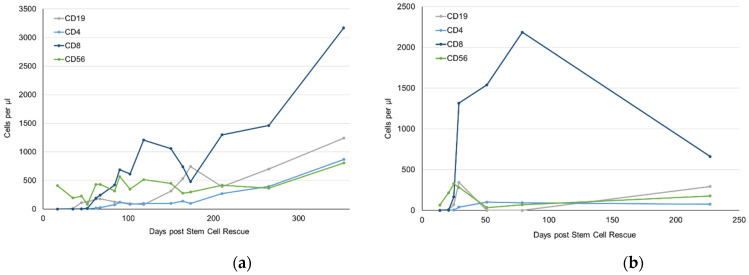
Immune reconstitution after immunoablative therapy in (**a**) case 1 and (**b**) case 2.

Both patients received GCSF until leukocyte reached 1/nL and showed an early leukocyte engraftment following IAT on day 9 and 10 posttransplant, respectively. Platelet engraftment was recorded as early as day 11 in both patients. In patient 1, immune reconstitution proceeded well, in particular after discontinuation of cyclosporine on day 46 posttransplant (Figure 5a, Table S2). In patient 2, EBV perturbed immune reconstitution profoundly, as we also administered rituximab (Figure 5b, Table S2).

**Figure 6:**
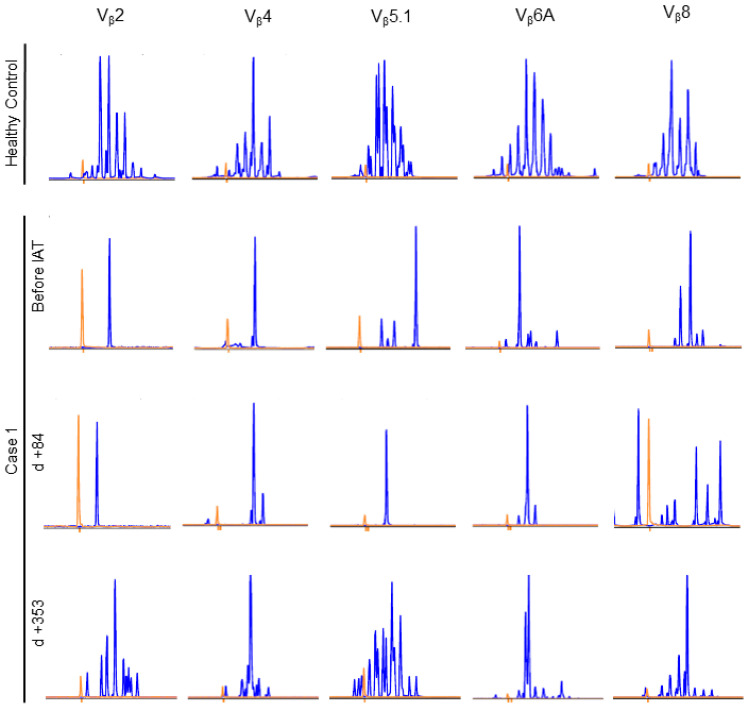
T cell receptor repertoire in peripheral blood assessed by V_β_-spectratyping in case 1.

One of the hallmarks of immune reconstitution is a broad T cell receptor repertoire, which can be assessed by V_β_-spectratyping of peripheral blood T cells. In contrary, severe graft-versus-host disease—and the accompanying immune suppression—often results in a pauciclonal V_β_ usage. While patient 2 still had too few T cells at last follow-up to get a meaningful result, we were able to analyze samples from patient 1. Prior to IAT, peripheral blood T cells displayed oligoclonal V_β_ usage under active cGvHD and the abovementioned therapeutic measures. However, soon after IAT with stem cell rescue and cessation of cyclosporine A on day 46 posttransplant, T cells developed polyclonal V_β_ usage. Nevertheless, even one year after therapy, T cell receptor repertoire still does not show normal diversity yet (Figure 6). Patient 2 has too few T cells for a meaningful V_β_-spectratyping (Figure 5b).

## 3. Discussion

The development of GvHD after HSCT is induced by numerous stimuli and responds well to treatment by steroids in the majority of cases. However, in the case of steroid-refractory GvHD, numerous salvage therapies have been employed [13]. Some patients, who progress from acute GvHD towards cGvHD, undergo several lines of immunosuppressive treatment for months and years, before they finally succumb due to organ failure or infectious complications. In a significant proportion of patients, the immunological network as a whole is so profoundly deranged that interference with selected signal transduction pathways is ineffective and tissue tolerance cannot be re-established [14,15]. In these desperate situations, a reset of the whole immunological network may be the only option to halt the progressive destruction of the recipient’s organs.

GvHD, as an allo-immune disease, shares some mechanistic and clinical features with severe autoimmune diseases, such as chronic inflammatory bowel diseases, scleroderma, and lupus erythematosus, to name a few [16]. Not surprisingly, well established treatment of theses autoimmune disease entities by biologicals also led to significant progress in GvHD. There are, however, entities, such as relapsing-remitting multiple sclerosis, where a reset of the whole immunological network by chemotherapeutic immune ablation and autologous stem cell rescue proved to be most effective [10]. We have had a similar experience in two pediatric cases with juvenile dermatomyositis [11].

These common immunological mechanisms and successful application of chemotherapeutic immune ablation with autologous stem cell rescue in autoimmune diseases led us to translate the approach into the allo-setting. To our knowledge there is only one letter to the editor by Arat et al. who presented the case of an adult patient with sclerodermatous SR-cGvHD. He received 4 × 50 mg/kg/day cyclophosphamide intravenously and ATG at 3 × 30 mg/kg/day prior to an CD34^+^ selected autologous stem cell rescue and showed partial response [17]. This approach is remarkable, because it is not clear upfront if stem cells can be harvested from a patient who had undergone an allogeneic transplant before and was treated heavily with myelosuppressive medication for a prolonged time under active cGvHD. The next risk is whether these cells engraft and reconstitute the complete hematopoiesis after transplant, as has been seen in mouse models. Moreover, contaminating T cells in the graft despite immunomagnetic sorting of CD34^+^ stem cells may convey the GvHD activity across the immune ablation.

To address these risks, we first chose a patient who had been transplanted from a matched sibling, so that we would have been able to obtain a second donation of stem cells in case she had no mobilized stem cells or these cells would not have engrafted. Because inevitably contaminating T cells will be present in the CD34^+^ selected graft, the leukapheresis was preceded by two courses of high-dose cyclophosphamide, which should ideally have depleted activated T cells from the circulation. Interestingly, both patients reported relief of certain GvHD symptoms already after commencement of the cyclophosphamide pulse therapy. We chose a very intense immune ablation, because we wanted to be sure to eliminate the adaptive immune system as profoundly as we could. The viral activations in both patients may be indicative for the intensity of this regimen.

Another concern when applying IAT to an HSCT recipient with an underlying malignant disease is whether or not the graft-versus-leukemia (GvL) effect may be compromised. As both patients have already been leukemia-free for more than one and a half years, relapse probability was reduced already. It is unclear, if there is a minimum of time required for the new graft to exert the GvL effect. The risk of relapse has to be pondered against the mortality and morbidity of SR-cGvHD in pediatric patients.

It is most important to learn how the immune system recovers after immune ablation and how we can assure that alloreactivity does not reoccur. In the autoimmune disease setting, this has been addressed in various publications [18,19]. Both of our patients showed rapid hematological reconstitution (Figure 5). However, the T cell compartment lacked behind significantly. T cell receptor repertoire was pauciclonal for more than one year in the first patient (Figure 6). In the second patient, T cell numbers were still under the lower age limit eight months after treatment, so that V_β_ spectratyping was not informative. This may be due to chemotherapeutic pretreatment and irradiation of thymic stroma, or to immunological damage to the thymus during GvHD and the treatment thereof [20].

Both patients faced a 5–year survival rate of only 25% [6]. Our decision was based on carefully weighing the risk of immune ablation against the risk of disease-related mortality. Both patients tolerated the treatment well, showed limited toxicity, and required support by only six transfusions of platelets and five erythrocyte concentrates on average. Interestingly, both patients showed a rapid reconstitution of hematopoiesis as to be expected from other autologous transplantations. This shows that allogeneic human HSC can be re-transplanted. While patient two was affected by an EBV reactivation and required rituximab, no critical infectious complications were encountered. Both patients clearly benefitted from the concept with one complete response and one partial response with ongoing improvement. The immediate and prompt immunological changes were realized by both patients after a disease course of two years in the first case and one year in the second case. Although sequelae will probably remain, e.g., avascular necrosis of the bone in patient 1, quality of life has improved vastly by increased mobility, ability to dress and undress, ability to swallow food etc.

In conclusion, immunoablative therapy and stem cell rescue is an attractive option for therapy-refractory, severe cGvHD. To avoid treatment sequelae, this kind of treatment may be considered earlier among the salvage therapies available. Definitely, more data is needed to achieve a sound risk-benefit-assessment of this therapeutic option in cGvHD.

## 4. Materials and Methods

### V_β_-Spectratyping

Genomic DNA (gDNA) was isolated from peripheral blood using QIAamp DNA Blood Mini Kits (Qiagen, Hilden, Germany) according to the manufacturer’s protocol. gDNA was stored at −20 °C until analysis. V_β_-spectratyping was performed by PCR amplification of CDR3 regions followed by fragment analysis, as has been in general described by Pannetier and colleagues [21]. Briefly, a total of 50 µL PCR reaction consisted of 5 µL 10× Taq DNA polymerase buffer, 2.5 mM MgCl_2_, 0.2 µL recombinant Taq DNA polymerase (all Invitrogen/ThermoFisher Scientific, Waltham, MA, USA), dNTP mix (200 µM each), 100 µg/mL BSA (both ThermoFisher Scientific, Waltham, MA, USA), 0.4 pmol forward primer, 0.2 pmol reverse primer (Metabion, Planegg/Steinkirchen, Germany), and 200 ng gDNA. The PCR mixture was amplified in a thermal cycler (T_ADVANCED_ by Biometra/Analytik Jena, Jena, Germany) with the following conditions: denaturation (95 °C for 5 min), amplification (95 °C for 30 s, primer specific annealing 56–58 °C for 30 s, 72 °C for 30 s; 35 cycles), followed by a final 72 °C elongation step for 10 min. Next, 2 µL of the fluorescent PCR products were mixed with 18 µL Hi-Di™ formamide, and 0.25 µL GeneScan™ 500LIZ™ Size Standard (both Applied Biosystems/ThermoFisher Scientific, Waltham, MA, USA) and denatured at 86 °C for 2 min in a thermal cycler (T_ADVANCED_ by Biometra/Analytik Jena, Jena, Germany). Fragment analysis by capillary electrophoresis was performed on a SeqStudio™ Genetic Analyzer using SeqStudio™ Genetic Analyzer Cartridges v2 (ThermoFisher Scientific, Waltham, MA, USA). Data analysis was carried out with GeneMapper™ software version 5 (ThermoFisher Scientific, Waltham, MA USA). Expected fragment lengths ranged between 245 and 274 bp (Table 1).

## Figures and Tables

**Table 1:** Forward and reverse primer for V_β_-Spectratyping; sequences adapted from [22].

Primer Forward	Modifi-Cation	Sequence	Annealing Temperature
V_β_ 2	6-Fam	AAC TAT GTT TTG GTA TCG TCA	56 °C
V_β_ 4	6-Fam	CAC GAT GTT CTG GTA CCG TCA GCA	56 °C
V_β_ 5/1	6-Fam	CAG TGT GTC CTG GTA CCA ACA G	58 °C
V_β_ 6a/11	6-Fam	AAC CCT TTA TTG GTA CCG ACA	56 °C
V_β_ 8a	6-Fam	CTC CCG TTT TCT GGT ACA GAC AGA C	56 °C
Reverse			
Jß 1.1		CTT ACC TAC AAC TGT GAA TCT GGT G	56 °C

## Supplementary Materials

The following supporting information can be downloaded at: https://www.mdpi.com/article/10.3390/ijms232315403/s1.
