# Supplementary material for: Immune Ablation and Stem Cell Rescue in Two Pediatric Patients with Progressive Severe Chronic Graft-Versus-Host Disease
Source: Int J Mol Sci. 2022 Dec 6;23(23):15403. doi: 10.3390/ijms232315403 (PMC9735744; doi:10.3390/ijms232315403)
Supplement: Supplementary file 1 [file ijms-23-15403-s001.zip › ijms-2029530-supplementary.pdf]

Supplementary Table S1. Patient characteristics in detail.

	Case 1	Case 2
Age, Sex	12 yrs, female	16 yrs, male
Diagnosis	Relapsed Pre-B ALL	common ALL, bcr/abl$^+$
Donor of initial HSCT	MSD (brother)	MUD (male)
Stem cell source	Bone marrow	PBSC
Conditioning	TBI, VP-16	TBI, VP-16, ATG
CMV serostatus (recipient; donor)	positive; positive	negative; negative
EBV serostatus (recipient; donor)	positive; negative	positive; positive
GvHD prophylaxis	Cyclosporine A	Cyclosporine A, methotrexate
cGvHD scoring before IAT	Skin: 3, joints and fascia: 3, lungs: 2, eyes: 1	Skin: 3, joints and fascia: 3, lungs: 2, eyes: 1
Karnofsky/Lansky before IAT	20	20
Echocardiography before IAT	Ejection fraction: 59%, fractional shortening: 30%	Ejection fraction: 71%, fractional shortening: 40%
Renal, liver and coagulation function before IAT (at admission to hospital)	Creatinine: 0.35 mg/dl, total bilirubin: 0.5 mg/dl INR: 1.1	Creatinine: 0.30 mg/dl, total bilirubin: 0.3 mg/dl INR: 1.1
IAT	Fludarabine (4 x 40 mg/m^2), cyclophosphamide (2 x 60 mg/kg), ATG (4 x 15 mg/kg)	Fludarabine (4 x 40 mg/m^2), cyclophosphamide (2 x 60 mg/kg), ATG (4 x 15 mg/kg)
CD34+ content of stem cell rescue	2.05 x 10^6/kg	2.4 x 10^6/kg
Engraftment after stem cell rescue	leucocytes > 1/nl since day +9, thrombocytes > 20/nl since day +11	leucocytes > 1/nl since day +10, thrombocytes > 20/nl since day +11
Response to IAT	complete response	partial response
cGvHD scoring after IAT	skin: 0; joints and fascia: 0; lungs: 2; eyes: 0	skin: 2; joints and fascia: 2; lungs: 2; eyes: 0
Karnofsky/Lansky scale after IAT	70	50
Virus reactivation after IAT	CMV	EBV
Echocardiography after IAT	Ejection fraction: 83%, shortening fraction: 50%	Ejection fraction: 60%, shortening fraction: 32%
Renal, liver and coagulation function after IAT (at discharge from hospital)	Creatinine: 0.39 mg/dl, total bilirubin: 0.4 mg/dl, INR: 1.1	Creatinine: 0.36 mg/dl, total bilirubin: 0.8 mg/dl, INR: 1.2

Supplementary Table S2: Peripheral blood lymphocytes immunophenotyping.

a) Patient 1

Day after/before ASCT	Lympho-cytes [nl^{-1}]	CD19 [nl^{-1}]	CD4 [nl^{-1}]	CD8 [nl^{-1}]	CD56 [nl^{-1}]
-119	1872	0	243	1123	168
-87	1690	0	254	946	135
-79	1664	0	333	1015	100
17	456	0	0	0	410
35	232	9	0	0	193
45	380	114	0	4	228
52	236	123	5	14	83
62	832	175	17	191	433
67	900	180	27	243	432
84	960	125	77	422	317
90	1496	120	120	688	568
102	1206	96	84	615	350
118	1980	79	99	1208	515
150	1961	314	98	1059	451
164	1722	534	138	740	276
173	1653	744	99	479	298
210	2451	392	270	1299	417
265	3045	700	396	1462	365
353	6210	1242	869	3167	807

b) Patient 2

Day after/before ASCT	Lympho-cytes [nl-1]	CD19 [nl^{-1}]	CD4 [nl^{-1}]	CD8 [nl^{-1}]	CD56 [nl^{-1}]
-167	1080	594	173	184	108
-104	1245	685	149	212	187
-11	189	23	45	51	51
-7	151	142	2	2	5
14	87	3	1	1	66
21	276	11	0	6	218
25	536	70	5	166	327
29	2024	344	40	1316	283
51	1692	0	102	1540	34
79	2350	0	94	2186	71
227	1273	293	76	662	178
